# Accuracy of Sonographic Renal Pelvic Diameter in Assessment of Renal Function among Patients with Ureteropelvic Junction Obstruction using Scintigraphy as a Gold Standard

**DOI:** 10.7150/ijms.100216

**Published:** 2025-01-21

**Authors:** Mahasin G. Hassan, Tasneem S.A. Elmahdi, Moudi Alotaibi, Enas Fallatah, Faten A. Nasser

**Affiliations:** 1 Department of Radiological Sciences, College of Health and Rehabilitation Sciences, Princess Nourah bint Abdulrahman University, Riyadh, Saudi Arabia.; 2 Department of Diagnostic Radiologic Technology, Faculty of Applied Medical Sciences, Taibah University, Al-Madinah Al-Munawara, Saudi Arabia; 3 Department of Radiology, Madina Maternity and Children Hospital, Al-Madina Al Munawara, Saudi Arabia.

**Keywords:** accuracy, renal pelvic diameter, renal function, ureteropelvic junction obstruction

## Abstract

**Objectives:** The study aimed to assess the diagnostic accuracy of sonographic renal pelvic diameter (RPD) in pediatric patients with ureteropelvic junction obstruction (UPJO) and its potential as a solo diagnostic tool. The study also looked for a clinically meaningful cutoff point in sonographic RPD, with the goal of optimizing sensitivity and specificity for discriminating between normal and impaired renal function in UPJO cases.

**Materials and methods:** The study, which took place at a maternity and child hospital, involved 75 children under the age of three who had been diagnosed with UPJO. Data was collected from 2020 to 2022 using both ultrasonography and renal scintigraphy, with cases diagnosed using only one modality being excluded.

**Results:** The analysis included descriptive and t-tests. The results demonstrated a significant difference in sonographic RPD between pediatric patients with normal and impaired renal function and with large and normal renal diameters. The average renal pelvis diameter was found to be 2.2 ±1.9 cm. The sensitivity and specificity of sonographic RPD for predicting impaired renal function in UPJO vary significantly across multiple cutoff points, underlining RPD's diagnostic potential.

**Conclusion:** The study concluded that combining two modalities (ultrasound and scintigraphy) improves results when the renal pelvic diameter surpasses 1 cm.

## Introduction

In recent years, ureteropelvic junction obstruction (UPJO) has become more frequently detected due to the increased sensitivity of ultrasound scans. The prevalence is high among neonates with hydronephrosis. UPJO is one of the most common causes of congenital urinary tract obstruction 1.

Usually, the result of UPJO is hydronephrosis. The critical point is that untreated conditions will lead to a loss of kidney function 2. The retention and stasis of urine, with insufficient clearance of bacteria from the urinary tract, is a significant factor for most urinary pathogens 3. One of the most common causes of renal failure in infants and children is obstructive nephropathy due to congenital hydronephrosis secondary to UPJO 4,5.

Renal pelvis diameter (RPD) is a significant indicator of serious conditions antenatally. Ultrasound is the chosen modality for assessing the renal pelvis in fetuses, neonates, and children 6. Ultrasound can measure the anteroposterior (AP) diameter at the maximal dilatation area of the intrarenal transverse plane 7. Among children, RPD differs according to age. It increases gradually from 3.1 mm in newborns to 5.5 mm at 18 years old 8. Measurements between 7 and 10 mm may indicate renal failure 9. The most effective diagnostic tools for investigating children with upper tract renal obstruction include ultrasonography and nuclear medicine 10,11. Ultrasonography is safe, noninvasive, easily accessible, and repeatable without radiation exposure 12, while renal scintigraphy provides information about renal perfusion and function 13.

Management and treatment decisions depend on the condition of the obstruction. Scans are repeated at different intervals, drainage is performed for patients with normal renal function, and surgery, such as pyeloplasty, is performed for impaired renal function 2,4,14. Most cases are resolved spontaneously 30 months after birth 15.

Glomerular Filtration Rate (GFR) measured by DTPA (diethylenetriamine pentaacetic acid) scan, and Effective Renal Plasma Flow (ERPF) measured by MAG3 (mercaptoacetyltriglycine) scan is used to assess the renal function. DTPA scan is mainly used to measure GFR directly, specifically targeting glomerular filtration. In contrast, MAG3 is predominantly used to evaluate effective renal plasma flow and tubular function rather than for direct GFR measurement. In clinical settings, these methods often complement each other. Research indicates that while DTPA is more suitable for direct GFR assessment, MAG3 can effectively estimate renal function, especially in tubular function and renal perfusion. Although each scan has its primary focus, both provide valuable insights into renal function 16, 17.

This study assessed the accuracy of RPD in renal function assessment with the gold standard, renal scintigraphy. The presence of a significant cutoff point for RPD could lead to a reduction in unnecessary imaging studies.

## Materials and Methods

### Research design

The study was a cross-sectional research design employing a quantitative research approach.

### Sample

The study included children younger than three years old diagnosed with UPJO and scanned using ultrasonography and renal scintigraphy, either by nuclear gamma camera scanning or positron emission tomography (PET). The study included all cases between 2020 and 2022, with a total of 75 cases using a convenience sampling method. Any case with vesicoureteral reflux, hydroureter, ureterovesical junction obstruction, posterior urethral valves and diagnosed with one modality (ultrasonography or scintigraphy) was excluded. The time interval between the ultrasound and scintigraphy was an average of two days.

### Data collection and analysis

This study retrospectively collected data from patients' records from the Picture Archiving and Communication System (PACS). All patients were well-hydrated for ultrasonography and scintigraphy. Renal ultrasonography was performed by trained sonographers using a GE Voluson E10 ultrasound machine. Renal ultrasound exams were performed by four sonographers, each with three years of experience in pediatric sonographic scanning. All sonographers worked under the supervision of five radiologists. Scanning was usually performed when patients were supine, using linear ultrasound transducers with an operating frequency of 5-10 MHz. The following images were acquired for both kidneys: longitudinal view: lateral, mid (with color Doppler) and medial, and transverse view of the upper, mid (with color Doppler), and lower renal levels. RPD was measured in the transverse section at the innermost maximum dilated part of the renal pelvis.

For renal scintigraphy, 29 patients were scanned using Philips - ADAC SKY Light nuclear gamma camera scanning, and 46 were scanned using Philips PET. The radiopharmaceutical drug used for nuclear scanning was 99mTc-DTPA (diethylenetriaminepentaacetic acid). For PET, the radiopharmaceutical drug used was 99mTc-MAG3 (mercaptoacetyltriglycine). The GFR and ERPF cut-offs used in the study reflect the renal function of the hydronephrotic kidney. The following parameters were assessed: site of obstruction (RT or LT kidney), rate of tracer (accumulation and washout), and renal function (either by measuring GFR or ERPF).

The hospital implements quality control measures to ensure adherence to the standardized protocol in renal scintigraphy. Two radiologists with more than five years of experience evaluated all images in this study. Moreover, the same protocol was used for all patients. A unified reporting format for documenting renal pelvis measurements was used for all patients. The hospital follows international standards for function interpretation. The GFR was considered normal when it was 28 ±26 mL/min/1.73m² for patients less than one year old, 43 ±28 mL/min/1.73m² for patients between 1-2.5 years old, and 72 ±40 mL/min/1.73m² for patients older than 2.5 years. ERPF measurement was based on Schlegel's method and classified as normal or abnormal. Schlegel criteria for younger children is considered normal when it varies between 200 to 600 mL/min/1.73m².

A data collection sheet that included all study variables was used to obtain data in patient records stored in PACS. The data sheet contains three parts. The first concerns demographic data (gender and age of the patient) and the cause of obstruction (congenital or acquired). The second part is related to sonographic RPD measurement. Data related to the classification of hydronephrosis was not available. The third part is related to the following renal scintigraphy parameters: site of obstruction (RT or LT kidney), rate of tracer (accumulation and washout; slow, rapid, or normal), and renal function (either by measuring GFR in nuclear scanning or ERPF in PET). The analysis was version 28 of the Statistical Analysis for Social Sciences for Windows (IBM Corp., Armonk, N.Y., USA).

### Ethical considerations

The study was approved by the Institutional Review Board (IRB) at Princess Nourah bint Abdulrahman University (no. 23-0126) and King Salman bin Abdulaziz Medical City (no. 22-072). There was no need for a consent form. The volunteer data were coded (patients' names were not mentioned) and used only for this study.

## Results

### Descriptive statistics

This study included 75 patients with UPJO with a mean age of 0.9 ± 1 years, the majority of whom were male (n=54; 72%), and the cause was congenital in most of the patients (n=73; 97.3%). Using ultrasound, the mean renal pelvis diameter was estimated to be 2.2 ± 1.9 cm, ranging between 0.1 cm and 10 cm. There was a very wide range in renal pelvic diameter measurements, 0.1cm to 10cm. However, these data were collected retrospectively from patient files, and the indications for what constitutes a normal renal pelvic diameter were not found.

Renal scintigraphy revealed that obstruction occurred in the left kidney in 33 (44%) patients and the right kidney in 42 (56%) patients. The rate of tracer accumulation was mainly rapid (n= 57; 76%), while washout was slow (n=63; 84%). Kidney size was enlarged in 38 (50.7%) of patients, while abnormal function was detected in 38 (50.7%) of patients.

### Inferential statistics

An Independent Sample's t-test was used to examine the RPD mean difference between the normal and abnormal renal function groups. The difference was considered significant (Table 1).

The mean RPD for individuals with normal renal function was significantly lower than the mean RPD for patients with abnormal renal function. A p-value less than 0.05 indicated that there is a statistically significant difference in mean RPD between patients with normal and impaired renal function. The observed difference in mean RPD values was not only statistically significant, but it may also have clinical significance, indicating that RPD can be used as a potential diagnostic marker to differentiate between normal and abnormal renal function in pediatric UPJO cases. The result suggested that abnormal renal function was associated with an elevated RPD in UPJO patients.

### Regression

This regression analysis (Table 2) examines the relationship between renal pelvic diameter (RPD) and abnormal renal function at three different cut-off points (1, 1.5, and 2). The results show that RPD is significantly associated with abnormal renal function at cut-off values of 1 and 2, with odds ratios of 4.3 and 2.7, respectively, indicating that larger RPDs substantially increase the likelihood of abnormal renal function. The relationship is strongest at cut-off 1, where the odds of abnormal function are over four times higher. However, at a cut-off of 1.5, the association is not statistically significant, suggesting it may not be as effective in predicting abnormal renal function.

The summary table (Table 3) shows how each cut-off for renal pelvic diameter (RPD) affects the ability to predict abnormal renal function (RFN). The main observation is that there is a trade-off between sensitivity and specificity as the cut-off increases.

- Cut-off 1.0 has the highest sensitivity (92.1%), meaning it is most effective in identifying cases of abnormal renal function. However, it has the lowest specificity (27.0%), indicating a high rate of false positives. This makes it a good threshold for minimizing missed abnormal cases but may lead to over-predicting abnormal renal function in patients with normal RPD.
- Cut-off 1.5 strikes a moderate balance, with a lower sensitivity (71.1%) and a moderate specificity (45.9%). The PPV (57.4%) and NPV (60.7%) are close, meaning it offers a more balanced prediction between true positive and true negative results, though neither is particularly high.
- Cut-off 2.0 has the highest specificity (73.0%), indicating it is best at identifying normal renal function (i.e., fewer false positives), but the lowest sensitivity (50.0%), meaning it misses a substantial portion of abnormal cases. The PPV is also the highest (65.5%), meaning that when it predicts abnormal function, it's more likely to be correct. However, its lower NPV (58.7%) means it is less reliable in predicting normal renal function.

## Discussion

Ultrasound is the most suitable method for urine tract examination, particularly for renal investigations 18. In detecting hydronephrosis, ultrasound has an overall diagnostic accuracy of 85.2% 19, and ultrasound has four classifications of hydronephrosis according to the Society of Fetal Urology (SFU) hydronephrosis grading system 20.

In renal failure (RF) cases, ultrasound has high accuracy in detecting obstructions 21, and it can differentiate acute from chronic RF 13. The ultrasound image showed reduced renal size, parenchyma thinning, and hyper-echogenicity in chronic RF. However, the exact diagnosis of the underlying chronic disease is not generally possible with ultrasound only 22. Renal scintigraphy remains the gold standard for assessment of split renal function 13. The radiotracers provide renal functional measures, such as glomerular filtration rate, effective renal plasma flow, tubular function, and renal blood flow 23. The study was conducted among children under three years old since UPJO never progressed after age two, and all subsequent procedures were performed until age three 24.

The study revealed that most UPJO cases were congenital. This aligns with the literature stating that congenital causes are the most common etiology 2. Similar to what has been found in previous studies, the results show that cases are more common in males than in females 25, 26, 27. Unfortunately, to date, there is no known justification for this difference. The study showed that all cases were unilateral, and no bilateral cases were among children under three years old. The literature has established that the left side is more affected than the right 2 and that congenital anomalies are high on the same side 28. The prevalence of the disorder in this study is high on the right side, which needs more studies to be confirmed and justified 29.

The standard deviations for RPD were quite large, even in the abnormal function group (2.7+2.1), indicating that several RPD values were measuring less than 1 cm. It's possible that some of these lower RPD values resulted from a previous issue, such as a febrile urinary tract infection (UTI). Febrile UTI is a prevalent infection in children that can lead to renal scarring and may result in long-term issues, such as chronic kidney disease 30. Additional studies could clarify the connection between RPD measurements and functional outcomes. It would be beneficial to examine the patients' histories of UTIs or other insults to understand the observed data better.

Different studies have disclosed the relationship between renal size and function. Ziauddeen *et al.* found that fetal kidney volume was related to minor increases in the estimated glomerular filtration rate (eGFR) in mid-childhood 31. Jovanović *et al.* proved that renal size in chronic kidney disease (CKD) patients is associated substantially with renal function 32. However, it is important to note that nuclear medicine or magnetic resonance urography assesses many cases of hydronephrosis without functional compromise, and hydronephrosis does not necessarily indicate abnormal renal function.

This study shows that the RPD mean difference between the normal and abnormal renal function groups is considerable. RPD was higher among patients with abnormal renal function. The findings reveal a significant association between RPD and abnormal renal function at the 1 cm and 2 cm cut-off values, with odds ratios of 4.3 and 2.7, respectively. This suggests that larger RPDs significantly raise the probability of experiencing abnormal renal function.

The cutoff value of 1 cm will help us include the largest number of cases with abnormal renal function. It had a higher sensitivity equal to 92.1%. This threshold effectively minimizes missed abnormal cases but may result in over-predicting abnormal renal function in patients with normal RPD. Patients with RPD of 1cm or less are likely to have a normal function, assuming the contralateral kidney is also normal because this corresponds to relatively mild hydronephrosis.

The cutoff value of 2 cm had a higher specificity of 73% (have fewer false positives), which will help exclude the largest number of cases with normal renal function. At this threshold, there is a strong likelihood of accurate predictions of abnormal function, while the lower NPV suggests reduced reliability in confirming normal renal function. A cut-off of 2 cm misses a few cases with hypofunction but will be more specific. The study performed by Botros *et al.* confirmed these results. Their study found that APD was significantly correlated with renal function and is an effective parameter for surgical decisions 33.

In the context of evaluating the accuracy of sonographic renal pelvic diameter in assessing renal function among patients with ureteropelvic junction obstruction, the use of biomarkers holds significant promise. For example, Paraboschi *et al.* have demonstrated the potential of urinary biomarkers in detecting damage associated with pelvic-ureteric junction obstruction, offering a non-invasive complement to traditional imaging methods 34. Similarly, Madsen highlighted the utility of urinary biomarkers in assessing hydronephrosis, suggesting that these biomarkers can provide valuable insights into renal function and damage 35. Incorporating these biomarkers could enhance the accuracy and overall diagnostic capability in assessing renal conditions beyond what sonographic measurements alone can offer.

The study's limitations included using two different protocols for renal scintigraphy, two different parameters to assess renal function, a small sample size, and the study's retrospective nature. To improve accuracy, using one protocol for renal scintigraphy, increasing the sample size, and conducting a prospective study could be beneficial. The possibility of other contributing variables, e.g., febrile UTI, is one of the study's limitations.

## Conclusion

Careful sonographic RPD had a high diagnostic value for renal function. The combination of two modalities (ultrasound and renal scintigraphy) increases the accuracy of results when RPD exceeds 1 cm and helps in therapeutic decision-making. Only 8% of patients with APRPD <1cm are likely to have decreased function and thus can safely be monitored with renal sonogram only in most cases. This will lead to a reduction in unnecessary imaging studies. Despite these results, clinical decisions should be made cautiously, considering the limitations of ultrasound, since overdiagnosis or underdiagnosis can lead to severe clinical consequences (such as unnecessary treatments). In the future, a prospective study overcoming the limitations of this study will improve and verify the study's results.

## Funding

The authors extend their appreciation to the Deputyship for Research & Innovation, Ministry of Education in Saudi Arabia for funding this research work through the project number RI-44-0881.

## Figures and Tables

**Table 1:** Comparison between RPD mean and renal function.

Renal Function	RPD Mean ± SD	t	*df*	P-value
Normal	1.799 ±1.55	-2.125	73	0.037*
Abnormal	2.711 ± 2.11			
Total	2.261 ± 1.90			

*Difference is significant

**Table 2:** Regression analysis

Predictor		B	Sig.	Exp(B)	95% CI for Exp(B)
Cut off 1	RPD	1.463	.038	4.321	1.08 - 17.23
	Constant	-1.204	.067	.300	
Cut off 1.5	RPD	.735	.131	2.086	1.60 - 12.15
	Constant	-.435	.261	.647	
Cut off 2	RPD	.993	.044	2.700	2.07 - 16.75
	Constant	-.351	.241	.704	

RPD = the size of the renal pelvic diameter categorized into normal and large.

**Table 3:** Summary table

Cut-off	Sensitivity	Specificity	PPV	NPV
1.0	92.1%	27.0%	56.5%	76.9%
1.5	71.1%	45.9%	57.4%	60.7%
2.0	50.0%	73.0%	65.5%	58.7%
